# Synchronous brain activity in newborn infants and their mothers during parent-led multimodal pain alleviation with skin-to-skin contact, breastfeeding and lullaby singing: Study protocol for a fNIRS hyperscanning study

**DOI:** 10.1371/journal.pone.0353856

**Published:** 2026-07-16

**Authors:** Mats Eriksson, Majja Lund, Mussie Msghina, Maria Reingardt, Alexandra Ullsten

**Affiliations:** 1 Faculty of Medicine and Health, School of Health Sciences, Örebro University, Örebro, Sweden; 2 Mental Health Clinic for Children And Adolescence, Primary Health Care, Region Örebro County, Örebro, Sweden; 3 Faculty of Medicine and Health, School of Medical Sciences, Örebro University, Örebro, Sweden; 4 Center for Clinical Research and Education, Region Värmland, Karlstad, Sweden; Niigata University, JAPAN

## Abstract

Protecting infants’ vulnerable brain during repeated painful procedures like blood-sampling and immunizations in early life is a top priority in neonatal health care. Use of parent-delivered pain management may safeguard the infant brain but at the brain level, it is still unclear what drives the analgesic effect of parent-led interventions such as skin-to-skin contact, breastfeeding, and the parent’s live singing. The objective of this study is to advance our understanding of the neural mechanisms behind parent-led pain management in newborn pain care. Twenty mother-infant dyads will be studied during a planned blood sampling venepuncture, with skin-to-skin contact, breastfeeding, and the mother’s live singing. The potential interbrain synchronous cerebral activation in cortical regions of interest will be registered using functional Near-Infrared Spectroscopy (fNIRS) hyperscanning. Secondly, registration of skin conductance, heart rate variability, and the mother’s gaze will be registered. Social and musical interactions will be studied using microanalysis, as will pain using the Behavioral Indicators of Infant Pain (BIIP). The Swedish Research Ethical Review Authority has approved the study (Dnr 2025-02283-01).

## Introduction

Today, we know that infants, including prematurely born infants, feel, experience and have a sensory memory of pain and that they are more vulnerable to the negative effects of pain than older children and adults [1–3]. Modern neuroimaging techniques combined with quantification of painful events show a clear positive correlation between higher neonatal pain exposure and functional and structural deviations in the growing brain [4,5]. It is also shown that a higher number of invasive procedures is associated with more sensory processing problems and altered hypothalamic-pituitary adrenal (HPA) axis function, which can be harmful for the infant’s developing nervous system and can divert energy from crucial brain development, at least up to school age, especially in girls [6,7]. In contrast, reducing pain in early life leads to more optimal brain growth and neurodevelopment [8].

Goksan and colleagues showed using functional Magnetic Resonance Imaging (fMRI) that infants can process and interpret pain in a manner similar to adults, and that almost all regions of the brain that are activated during acute pain in adults are also activated in healthy full-term newborn infants [1,9,10]. This suggests that infants have the capacity to experience the affective dynamics of pain in the same way as adults [1]. The same group has also shown using fMRI that the descending pain modulatory system is fully functional at birth in term-born healthy infants, similar to that observed in adults [9]. This may suggest that in the term newborn infant, brain regions involved in descending pain modulation may influence the dampening, alteration, and alleviation of pain experience, as well as the modification of pain behaviour.

The neural mechanisms underlying emotion and social cognition as well as early sensory and recorded music processing in infants, have previously been investigated using functional Near-Infrared Spectroscopy (fNIRS) [11]. In one fNIRS study, preterm infants repeatedly listened to recorded Western art music, and the results showed significant hemodynamic changes in the left superior temporal gyrus in response to variations in the recorded music (timbre, dynamics, and rhythm). The authors concluded that infants’ auditory cortex demonstrate functional maturity in processing multiple dimensions of recorded musical input and the results support the evidence of synchronous large-scale brain networks involved in musical perception [12]. fNIRS studies investigating newborn infants’ brain responses to maternal speech, showed that infants are highly responsive to their mother’s emotional prosody with significantly enhanced functional connectivity within the bilateral frontotemporal network, as well as increased neural activity in the right superior temporal gyrus during the mother’s affectively expressive speech, especially positively charged mother-infant emotional interactions [11,13]. In previous studies, fNIRS has demonstrated that tactile stimulation, especially through skin-to-skin contact, significantly activates the frontal, somatosensory, and motor cortices in preterm neonates supporting emotional regulation [14]. It is reasonable to deduce that infants from birth and at brain level are responsive to their psychobiologically attuned parents’ multimodal and multisensory visual-facial, auditory-prosodic, and tactile-gestural nonverbal pain-alleviating interventions, especially positively valenced nonverbal communications, such as the parent’s live infant-directed singing [15], skin-to-skin contact [16], and breastfeeding [17].

### Synchrony for pain alleviation

Synchrony is described as a complex social coupling process of two or more individuals in the here-and-now, and interbrain synchrony refers to an alignment of brains between two individuals [13]. Early stressful ruptures of the infant-parent emotional attachment bond that are not routinely followed by repair interactions, occurring, for example, during painful procedures poorly alleviated and performed without the parent’s protective comfort and love, will cause disturbances in the synchronization of the dyad’s interpersonal psychobiological systems [13]. Lack of synchrony with the parent is stressful for the infant, and stress may exacerbate a painful experience. In a stressful and painful situation, such as the blood sampling procedure, the infant seeks proximity to the parent, not just physical closeness but an emotional mind-body proximity where a right brain-to-right brain nonverbal communication is shared between the infant’s and parent’s minds [13]. The empathic psychobiologically attuned parent will intuitively, nonverbally, and interactively regulate her infant through social biofeedback maximizing positive and minimizing negative affect to maintain interbrain synchrony in the dyad. Right-lateralized interbrain synchronization, i.e., a synchronization between the right temporoparietal cortex of the infant brain and the right temporoparietal cortex of the parent’s brain, is embedded in the parent-infant biopsychosocial interplay associated with synchronous multisensory stimulation [13]. Shared positive affects specifically activate the parent’s and her infant’s right temporoparietal junction, known as the central node of the social brain, involved in regulation between and within brains, minds, and bodies [13]. Since this region of interest, the right temporoparietal junction and surrounding temporoparietal cortex, sits on the cortical surface, it is accessible to fNIRS and hyperscanning techniques.

Stress, which is a dominant element in pain, is defined as “an asynchrony in an interactional sequence” [13]. When a period of stress is followed by a repair period of synchrony, this provides regulation and recovery in the parent-infant dyad. Emotion regulation is key for pain alleviation [18,19]. The dyad’s nonverbal reciprocal moment-to-moment right brain-to-right brain emotional protocommunications of face, prosody, and gestures, will enhance synchronization followed by mutual regulation of the dyad’s emotional states which will give the infant and the parent a sense of pleasure and vitality [13]. Regulation occurs when the mother-infant dyad is synchronized on all levels from neural activity to physiological states, such as heartbeat rhythm, to pupil size, to behavioural facial expressions, reciprocal vocalisations and body postures [13]. It is theorized that synchrony consists of biopsychosocial reciprocal processes, including co-regulation present in a mother-infant nonverbal protocommunication where both individuals adjust their social attention, and accelerate their arousal to each other [13].

Using hyperscanning with functional Near-Infrared Spectroscopy (fNIRS) [20] will enhance our understanding of the multimodal and multisensory biopsychosocial parent-led pain alleviating methods. Hyperscanning illuminates the social interaction between two nonverbally communicating brains [13]. In this hyperscanning study, based on previous research, we deduce that parent-led pain alleviating interventions, with bidirectional visual-facial (mutual gaze), auditory-prosodic (live parental singing), and tactile-gestural (affectionate touch, skin-to-skin contact and breastfeeding) nonverbal communications, will activate interbrain synchrony in the dyad helping the infant reach and maintain homeostasis and a state of regulation before, during and after the painful procedure. The proto-musical elements of parental infant-directed singing are ideal for arousal regulation and affect coordination as well as for affect sharing between parent and infant, and the parent’s live singing provides a contingent responsivity and continuum in the regulation of the infant’s arousal systems [18]. Hence, positively charged interbrain synchronization has the capacity to dissolve a negative painful affective spiral.

In moments of interpersonal synchrony, previous research shows that maternal live infant-directed vocalizations as well as skin-to-skin contact promote stabilization of physiological and behavioural parameters regulating the infant’s autonomic nervous system, heart rate, and respiratory rate [21].

Heart rate variability (HRV) is a non-invasive measure of cardiac autonomic control giving information on the sympathetic and vagal modulations evoked by pain [22]. In newborn infants HRV is considered an indicator of the maturity of the autonomic nervous system [23]. During the first week of life, both total, low-frequency (LF), and high-frequency (HF) HRV following acute pain showed inconsistent results among full-term infants. The LF/HF ratio decreased in response to acute pain and was the only consistent measure [24]. An increase in skin conductance (SC) reflects the infant’s arousal intensity and level of stress in response to a painful procedure. Skin-conductance, also named Galvanic Skin Response (GSR) or Electrodermal activity (EDA), is a measure of the sympathetic nervous system’s response to stress and pain [25,26]. An increase in the number of SC peaks per second, as well as the area under small peaks and the area under large peaks, indicates a higher stress level.

Mutual gaze is essential for the interbrain synchrony in the right temporoparietal cortex which responds to visual, auditory, and tactile stimuli [13]. Adult gaze serves as a pivotal social cue in early development, with infants demonstrating marked sensitivity to its presence and directional orientation [27]. In a study of vaccination of 2-month-old babies, it was found that the mother-to-infant gaze increased if the mothers sang live to their infants [28]. The infant’s facial actions, especially movements in the mouth area, are thought to be important behavioural responses to pain, helping infants to communicate their affective state with their caregivers. Recent studies on neonatal pain assessment have used eye-tracking device to evaluate where the observer looks during pain assessment of the infant [29–32]. In one study, when adults with and without a health care profession assessed pain in infants from looking at pictures of infants’ faces, the health care professionals more often correctly identified the infants’ pain by looking repeatedly and longer at the nasolabial furrow feature in the faces [29]. A similar study design was used comparing the correlation of visual tracking between health and non-health professionals when observing newborn infants’ face images at rest and during a painful procedure [31]. Health care professionals focused more on the infants’ mouths and the nasolabial furrows and had fewer visual fixations on the infants’ eyes compared to non-health professionals [31]. Silva and colleagues [30,32], investigated health care professionals’ gaze when in the first study assessing pain from images of newborn infants at rest and during a painful procedure and, in the second study, when assessing newborn infants pain expressions during a live heel puncture procedure. In the first study, the paediatricians’ gaze fixations were greater in the infants’ mouth and forehead than in the nasolabial furrow [32]. Results from the second study showed that most paediatricians focused their visual attention on the infant’s face, especially on the lower face, than on the infant’s hands [30]. Little is known, however, about how parents look at their infants while interacting with them during a painful situation. Social mutual gaze is just one attribute through which the psychobiologically attuned mother perceives and resonates with the infant’s pain experience.

### Parents alleviate infants’ pain responses

It is crucial for an infant in a painful situation, as well as for forthcoming painful experiences, that her/his parent is emotionally available, attuned to the infant’s state, and attuned to moment-to-moment changes of the infant’s affects, capable of noticing signals and able to regulate and share the infant’s states before and during the pain experience [18]. However, it is not yet established which brain processes are involved in parent-led pain management and which brain functions are coupled and synchronous in the parent and infant brain when a parent sings in combination with skin-to-skin contact and breastfeeding during a painful procedure.

In an ongoing music therapy study, in a non-painful context, fNIRS is used to investigate the synchronization of cerebral activation between the infant and the parent, as well as between the infant and the music therapist while she sings lullabies accompanied by a vibro-acoustic monochord during parent-infant skin-to-skin contact [33,34]. In a randomized controlled study, using fMRI, the infant-directed live singing performed by a music therapist had beneficial effects on very preterm infants’ brain network connectivity in cognitive, socio-emotional, and motor functions [35]. Prior research has emphasized the importance of including parental vocalizations in neonatal pain management, showcasing its ability to improve the infant’s physiological stability, emotional regulation, and attachment to carers including mitigating the infant’s pain responses and decreasing parents’ anxiety [36].

The interaction between mothers and their infants in a painful context [37,38] has previously been studied with fNIRS and hyperscanning [20,39]. Bembich et al. showed synchronized cortical activation between the mother and her newborn infant while the mother, without holding the infant, observed heel prick blood sampling on her infant [40].

To our knowledge, no previous research has used fNIRS and hyperscanning to explore interbrain synchrony in infants and their mothers during parent-led multisensory and multimodal pain alleviation. It is important to develop evidence-based in-depth knowledge to better inform neonatal care on how and why combined parent-led pain management should be promoted and implemented. This will increase parents’ possibilities to protect their infant’s brain from pain during the many painful procedures early in a child’s life.

Protecting infants’ vulnerable brain during repeated painful procedures like blood-sampling and immunizations in early life is a top priority in neonatal health care. Parents are, and should be, a fundamental resource in infant pain management, for both healthy and hospitalized infants [41,42]. With interbrain synchronized regulation in the mother-infant dyad, the use of parent-delivered pain management may safeguard the infant brain during a painful procedure but, at the brain level it is still unclear what drives the analgesic effect of parent-led interventions.

## Materials and methods

The overall objective of this project is to advance our understanding of the neural mechanisms behind parent-led pain management in newborn pain care. Understanding at the brain level the mechanisms that modulate the effects of parental interactions with skin-to-skin contact, breastfeeding, and parents’ live singing on pain perception in neonates, could also provide insight into pain learning and protective actions against repeated pain exposure for the parent-infant dyad.

### Hypotheses

1. Changes in oxygenated haemoglobin (Oxy-Hb) and deoxygenated haemoglobin (Deoxy-Hb) in the prefrontal, posterior frontal, parietal and temporal areas in both hemispheres of the brain in mothers and infants will be synchronously activated, during an intervention with skin-to-skin contact, breastfeeding, and parent’s live singing during venepuncture for blood sampling.
2. Corresponding synchrony will be seen in heart rate variability and skin conductance activity.
3. The neurophysiological responses are correlated to the infant’s pain reaction, parent’s gaze, and social and musical interactions.

### Sample, setting and procedure

#### Study participants*.*

Twenty mothers and their healthy newborn infants will be recruited among birthing mothers in Örebro county, Sweden. Inclusion criteria: healthy mothers above 18 years of age, able to understand Swedish or English language, having had a vaginal delivery; infants with gestational age above 37 + 0, birth weight above 2 500 g, and Apgar score at 1 minute above 7. Exclusion criteria: infants who received analgesics or sedatives within 24 hours before the blood sampling, or who failed at the newborn hearing screening test. The number of mother-infant dyads was motivated from previous studies using fNIRS during heelstick (Bembich 2022: 16 dyads [40]; Bembich 2018: 20 dyads in each group [43]), and from the above-mentioned ongoing music therapy study in Switzerland [33,34]. The sample size was determined using a Minimum Effect Size of Interest (MESI) analysis (G-Power 3−1, Heinrich Heine University, Düsseldorf, Germany) [44]. Effect size 0.35, alpha 0.05, beta 0.80, 7 repeated measurements, revealed a sample size of 11 dyads. To compensate for attrition, we will recruit 20 dyads and the pilot test with two dyads indicates that both recruitment and the recording procedures are feasible and easily tolerated by the mother and the infant. The initial recordings will help in optimising all recordings to get an analysable sample of at least 11 dyads. The mother and infant will be studied during the ordinary blood sampling for metabolic screening, done by venepuncture, i.e., no extra pain will be inflicted.

#### Procedure*.*

**Fig 1:**
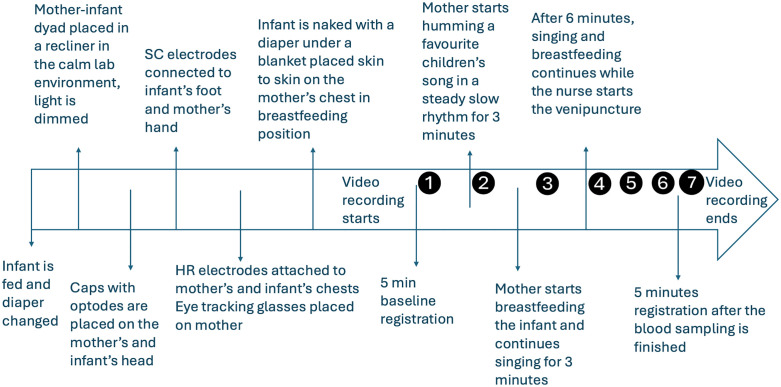
Timeline showing the procedures in the test-setting. The numbers indicate timepoints for markers in the recording equipment and subsequent data analysis: 1 start of recording, mother and infant sit skin-to-skin, 2 start of singing, 3 start of breastfeeding, 4 the nurse touches the infants arm to start searching for a vein, 5 punction of the skin, 6 the needle is removed, 7 end of recording. SC = skin conductance activity, HR = heart rate.

Testing will be conducted in a calm lab environment, and the infants will be fed and have a dry diaper before the equipment is applied. Before testing, the researchers will guide the mothers verbally on how to sing a song of their own choice, which can be a song of kin that is a personalized song meaningful to the mother aiming to foster bonding, reduce stress, and improve physiological stability in infant and mother, or a traditional children’s play song. The mother is instructed to hum and sing with or without words in a positive, joyful expressive affect in her prosody, using a steady, repetitive and predictable pulse in her song, singing with a smiling exaggerated expression in her face to capture her infant’s attention, striving to establish a mutual gaze and eye-to-eye interaction with her infant, accentuating the singing rhythm in her affectionate touch (if applicable), overall to sensitively adjust and match her singing style and her multimodal communications (auditory-prosodic, visual-facial, and tactile-gestural nonverbal communications) to her infant’s cues, responses, and state, aiming to create a positive affect in the dyad with her interactive multisensory stimulation and regulation. The equipment is first attached to the mother and then to the infant, ensuring good signal quality and minimizing disturbance of the infant. To synchronise measurements (fNIRS, SC, HRV, eye tracking, and video) we will verbally announce the time-points 1–7, and simultaneously manually mark the events in the equipment with keyboard markers (Fig 1). The mother and infant will then sit together in a comfortable chair in skin-to-skin position for baseline registration lasting 5 minutes (1). When we have steady-state signals and a stable baseline, the mother will start singing (2) and she continues singing for 3 minutes. After that, she offers the infant to breastfeed (3). After another 3 minutes, or until the baby has a good grip on the breast, a nurse will search for a vein (4) and perform the venepuncture (5), while the mother continues to sing and breastfeed until blood collection is ready (6). Finally, 5 minutes of signal will be registered after the blood sampling is finished (7). Before regular data collection begins, two pilot tests were conducted to calibrate the equipment and time intervals.

### Measurements and instruments

**Fig 2:**
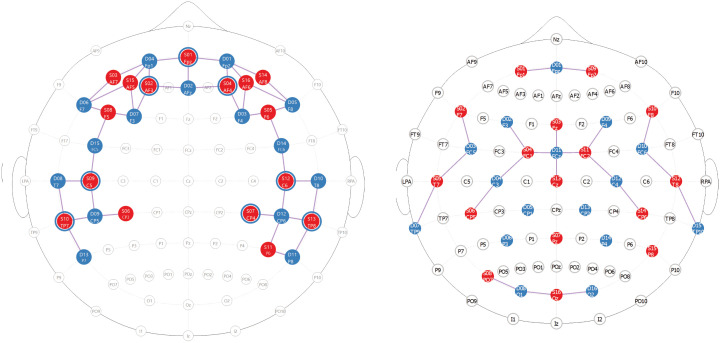
Infant (left) and mother (right) montage of fNIRS-optodes. Red = transmitter, blue = detector, circle = short channel. The mother montage is set to cover the following regions of interest: prefrontal, posterior frontal, parietal, and temporal regions of the brain including the right and left temporoparietal junction. The infant montage will cover the whole head and thus the same regions.

*Cerebral hemodynamics* will be recorded using functional Near Infrared Spectroscopy (fNIRS). Both mother and infant will wear head caps with optodes (transmitters and detectors) connected to a NIRSport2 data acquisition module (NIRx Medical Technologies, Berlin, Germany). The optode montages are designed in NIRSite 2.0 software (NIRx Medical Technologies) and imported into the data acquisition software Aurora fNIRS (NIRx Medical Technologies). The optodes in the mother montage are set to cover the following regions of interest (ROIs): prefrontal, posterior frontal, parietal, and temporal regions of the brain including the right and left temporoparietal junction, to capture auditory and visual functions as well as emotional and social regulation activities between and within brains [13,40,45]. The infant cap montage will cover the whole head, i.e., synchrony in the same ROIs will be registered on both subjects. No previous findings on synchrony during mother-led pain alleviation exist but Bembich et al. found activation in mothers’ parietal cortex and infants’ superior motor/somatosensory cortex when the mother’s observed a heel prick [40]. On both mothers and infants, we will use 16x16 channels set-ups following the 10–20 EEG placement system [46] (Fig 2) and the mother montage will include short channels to compensate for artefacts from superficial blood flow.

Oxygenated haemoglobin and deoxygenated haemoglobin (Oxy-Hb & Deoxy-Hb) will be registered for all channels in real-time using Aurora fNIRS and stored on a computer. The sampling rate will be set between 5 and 10 Hz using multilevel illumination.

Electrodes for registration of heart- and respiratory rate in the mother will be connected via a NIRxWINGS module (NIRx Medical Technologies) and registered in Aurora fNIRS. The infant will be attached to ECG-electrodes connected to a patient monitor (Philips IntelliVue X3, Eindhoven, NL).

From the heart rate (HR) registration heart rate variability (HRV) will be calculated. Electrodes for registration of skin conductance activity (SC) will be attached to the palmar side of the hand of the mother and connected via a NIRxWINGS module and registered in Aurora fNIRS. The infant will wear electrodes to record SC on the sole of the foot. The signal will be registered in GSR recording software (MedStorm, Oslo, Norway).

Two video cameras will be used, one to record the mother-infant interaction, later used for microanalysis of the live singing and social interactions [47], and one directed on the infant’s face, for later pain-scoring with the Behavioral Indicators of Infant Pain (BIIP) [48]. The mother will wear Tobii Pro 3 eye-tracking glasses to record her gaze at the infant [27].

### Data management plan

All participants will be given a study-ID to preserve confidentiality. The key code will be stored in a secure way, separate from all other study data. All measurements, including neurophysiological data and video recordings, together with characteristics of the participants will be stored on a secure server, with access only for the researchers.

### Data analysis plan

Recorded data will be analysed at the following specific time points, that may vary slightly due to signal quality but will be synchronized for mother and infant (fig 1):

1. baseline, when the mother and infant are settled comfortably, before the mother starts humming, approximately 2 minutes and 30 seconds after the marker,
2. when the mother has started humming, approximately 2 minutes after the marker,
3. when the mother has established active breastfeeding, approximately 2 minutes after the marker,
4. when the nurse is searching for a vein, approximately 30 seconds after the marker,
5. directly after the venipuncture needle penetrates the skin,
6. approximately 30 seconds after the blood sampling is finished, and
7. before data collection is finished, approximately 2 minutes and 30 seconds after the blood sampling is finished.

#### fNIRS data analysis.

fNIRS data will be analysed using Satori fNIRS analysis software (NIRx Medical Technologies). For the preprocessing, we will perform channel rejection either based on CV (coefficient of variance from other channels) or SCI (scalp coupling index). We will apply temporal filtering with a bandpass at 0,01–0,5 Hz, and linear detrending to avoid eventual shift in the data. We will perform motion correction and spike removal using TDDR (temporal derivative distribution repair) and remove physiological noise with SSR (short channel regression). We will calculate OxyHB and deoxy-Hb at the specified time points 1–7 and regions of interest. The analysis of the interbrain neural syncrony will follow the method described by Nguyen et al. [20], using Wawelet Transform Coherence (WTC).

#### HRV data analysis.

From the heart rate registration, we will calculate the low frequency / high frequency (LF/HF) rate of HRV, as a measure of sympatethic and parasympatethic contribution [24] at the time points indicated in 1–7.

#### SCA data analysis.

SCA magnitude and frequency will be calculated in Satori (mother) and MedStorm software (infant), at the specified time points 1–7.

#### Assessment of infant pain.

Assessment of pain signals in the infant will be done by scoring from the video recordings of the infant’s face. BIIP will be used, which scores infant state, five facial expressions and two hand actions [48].

#### Assessment of video recordings.

Social and musical interaction between the mother and the infant will be analysed with in-depth analysis of video footage and eye-tracking recordings [47]. There is a long tradition in developmental psychology research of studying parent-infant moment-to-moment interactions and communication using video recordings and microanalysis. Following the tradition, the researchers in this study will perform a hand-coded, second-by-second microanalysis of the naturalistic mother-infant moment-to-moment musical, vocal, verbal and non-verbal communication. In the microanalysis, researchers will log micro-interpersonal, interactive processes and details, as well as subtle occurrences and changes in the mother-infant musical and non-musical behaviors in relation to the parent-led pain management, the skin puncture and contextual elements in the environment. Events that are interesting to look for include those related to singing, such as changes in song, pitch, key, volume, or tempo. Events that have an impact on the infant’s pain behaviour, on infant’s overall behaviour/reactions, on the parent’s reactions, on infant’s state, including regulatory mother-infant behaviors will also be logged into an excel sheet as described in Ullsten et al., 2017 [47]. Validity will be secured through a thorough and systematic process, analysing each of the videos until no new details will be discerned. Validity will also be secured through an inter-coding peer review process between the researchers.

#### Statistical analyses.

The primary hypothesis, interbrain neural synchrony, will be analysed using General Linear Mixed Models (GLMM) and WTC [20] for both Oxy-Hb and Deoxy-Hb. After preprocessing of the signals, an interpersonal comparison will be performed for each region of interest and each time point, within each dyad. The false discovery rate (FDR) will be set to 0.05.

For the secondary outcomes, SC and HRV, synchrony will be analysed at the time points within each dyad before calculating means or medians on the group level. All statistical analyses will be done using parametric statistics (means, standard deviations, t-test or ANOVA) for data that are normally distributed and non-parametric statistics (median, inter-quartile range, Mann-Whitney or Kruskall-Wallis) for data that are not normally distributed. Chi-square test will be used to compare group distributions.

### Status and timeline of the study

All technical equipment is in place, and the fNIRS-montages are prepared in Aurora. Ethical approval is obtained.

- Spring 2026 Recruitment of study participants starts
- December 2027 Finalizing recruitment and data collection
- 2028 Data analysis and publishing

### Safety and adverse events

Parent-led pain management is safe and used in clinical routine. We do not expect any adverse events during the study, but if such occurs, they will be noted and handled accordingly. The data collection will be performed at the hospital’s brain laboratory under the surveillance of trained neonatal personnel.

### Research ethical considerations and declarations

The pain stimulus in the study is a routine blood sample for metabolic screening, i.e., no extra pain will be inflicted on the infant in the course of the study. The mother will use evidence-based pain alleviation methods, and her presence will be calming for the infant. The parent-led interventions in the study are non-pharmacological, safe and have no known side effects. The procedure, including attaching the optode caps and baseline registration will take about 30 minutes and will take place in a calm lab environment. Parents will receive both written and verbal information about the study. Written informed consent will be acquired from all parents, and all participants will have the right to withdraw at any moment. The Swedish Research Ethical Review Authority has approved the study (Dnr 2025-02283-01).

## Discussion

Our research group has studied parent-led pain relief in several studies: breastfeeding [49], skin-to-skin contact [16], and lullaby singing [15,50]. We have recently published results from a large randomized controlled multi-centre trial using all three parent-led interventions in combination showing that parent-led pain management combining skin-to-skin contact, breastfeeding, and parents’ live lullaby singing is a feasible and safe intervention with potential pain alleviating properties offering the parents a strong sense of meaningfulness and stress relief [51]. In our previous studies, we have used PIPP-R [52], skin conductance and other systemic physiological parameters [16,26,52], and NIRS/fNIRS [16,52,53]. Continued research is needed to deepen the knowledge of non-pharmacological parent-led interventions in neonatal pain care exploring what drives the analgesic effect of skin-to-skin contact, breastfeeding, and parents’ live singing. This hyperscanning study is a new, cutting-edge research project which will potentially advance research in numerous domains; neonatal pain research, music science, neuroscience, nursing science and music therapy science. To objectively assess pain and pain alleviation in the infant-parent dyad is a complex endeavor, as pain is an intertwined process of intrapersonal and interpersonal biopsychosocial dynamics and even emotionally contagious interactions [18]. Combining fNIRS and hyperscanning may potentially offer a more objective, neuroscientific method to evaluating pain perception and pain management by capturing real-time changes in cerebral hemodynamics.

Synchronization is a key feature of mother-infant turn-taking interactions, embedded in their reciprocal nonverbal visual-facial, auditory-prosodic, and tactile-gestural protocommunications [13]. We deduce that synchronic interbrain regulation in the dyad plays a key role in minimizing the infant’s pain experience by implicitly sharing their conscious and unconscious affective and emotional states in the dyad, and that the parent, the mother in this study, is mediating the pain relief when interactively downregulating negative effect and stress and upregulating positive effect in a loving playful musical synchronous relationship. Due to its temporality and multimodal expression, synchrony is challenging to examine objectively. To improve the analysis quality of synchrony, pain and pain alleviation, research suggests an integrative approach combining clinical observation and engineering techniques [9,54]. Interbrain neural synchrony in the mother-infant dyad has not previously been measured with fNIRS and hyperscanning in connection with a routine painful procedure and in association with combined parent-led pain reducing methods. This project is the start of a new path in family-centred non-pharmacological neonatal pain research, using fNIRS and hyperscanning combined with systemic physiological parameters, to investigate the dyadic feedback loop with co-regulation in the infant’s and parent’s brains during a painful procedure and parent-led pain management.

### Limitations

fNIRS and hyperscanning are non-invasive, mobile, and relatively motion-resilient brain imaging techniques suitable for naturalistic interactive social mother-infant observations [11]. However, potential technical, analytical, and conceptual challenges are legion in this research study. Synchronizing data across multiple recording devices is a crucial endeavor that will be managed comprehensively by the research group. Other pitfalls that will be carefully monitored by the research group are movement artefacts, poor optical coupling and data quality, as well as data attrition. Due to high equipment costs and time-consuming preparations, most fNIRS hyperscanning studies, including this project, are characterized by small sample sizes and single-center designs, which affects applicability and transferability of results [11]. As mentioned, fNIRS measures only the superficial cortical regions, and with a limited number of channels available per person this will reduce the options to capture comprehensive brain activity; a disadvantage when exploring intricate interbrain social interactions such as those in a mother-infant dyad. In addition, internationally standardized neonatal protocols for data processing and analysis in fNIRS hyperscanning is currently lacking, which might impede reproducibility across studies [11].

Yet, while facing the technical and analytical challenges, the results from this fNIRS hyperscanning project will hopefully advance both theoretical understanding and clinical practice in neonatal pain management. Because, despite a growing body of evidence supporting parent-led pain management, health care professionals still don’t prioritize parent involvement in infants’ pain care. Research needs to systematically add scientific knowledge on what drives the analgesic effect of parent-led pain management to improve the parents’ possibilities to protect their infant’s brain from pain during the many painful procedures early in a child’s life.
