# Supplementary material for: Synchronous brain activity in newborn infants and their mothers during parent-led multimodal pain alleviation with skin-to-skin contact, breastfeeding and lullaby singing: Study protocol for a fNIRS hyperscanning study
Source: PLoS One. 2026 Jul 16;21(7):e0353856. doi: 10.1371/journal.pone.0353856 (PMC13375031; doi:10.1371/journal.pone.0353856)
Supplement: Attachment — Submitted filename: Response to Reviewers.docx [file pone.0353856.s003.docx]

**Response to the editor’s and reviewers’ comments**

Thank you for the valuable comments on our manuscript. We have addressed them in the marked-up manuscript copy, and in the table below. Since the reviewers asked for many clarifications on rationale and methods, we have re-written some sections, rather than just inserting smaller sections and sentences. Since the concept synchrony was not previously clearly described in the manuscript, we have now included a more detailed framing of the concept synchrony and connected brain areas. We believe this makes the text more readable. We have highlighted the new parts of the text.

Sincerely, for the authors

/Mats Eriksson & Alexandra Ullsten

| **Academic editor** |  |
| --- | --- |
| 1. Please ensure that your manuscript meets PLOS ONE's style requirements, including those for file naming | Thank you, we have been careful to follow the instructions. |
| 2. In the online submission form you indicate that your data is not available for proprietary reasons and have provided a contact point for accessing this data. Please note that your current contact point is a co-author on this manuscript. According to our Data Policy, the contact point must not be an author on the manuscript and must be an institutional contact, ideally not an individual. Please revise your data statement to a non-author institutional point of contact, such as a data access or ethics committee, and send this to us via return email. Please also include contact information for the third party organization, and please include the full citation of where the data can be found. | We believe this is a misunderstanding. As this is a study protocol, no data are available at this time. However, we have a data contact point at our university and will provide the requested information. |
| 3. Your ethics statement should only appear in the Methods section of your manuscript. If your ethics statement is written in any section besides the Methods, please delete it from any other section. | We have moved the ethics statement to the Methods section. |
| 4. Please upload a new copy of Figures 1 and 2 as the detail is not clear | We have uploaded revised figures, following the instructions. |
| 5. If the reviewer comments include a recommendation to cite specific previously published works, please review and evaluate these publications to determine whether they are relevant and should be cited. There is no requirement to cite these works unless the editor has indicated otherwise. | We thank the reviewers for some useful suggestions of references, and have included them when appropriate. |
| **Reviewer #1** |  |
| 1. The research question outlined is expected to address a valid academic problem or topic and contribute to the base knowledge in the field.  The study protocol tackles a relevant research question, and the experimental hypotheses are clearly stated. However, while I understand the logic behind the choice of measuring HRV and GSR in this population, the rationale behind the choice of exploring interpersonal neural synchrony during infant pain management remains overall unclear. The authors should better describe the rationale that motivated the inclusion of interpersonal neural synchrony measures in their study. | Thank you.  The main focus of this study is to investigate synchronous brain activity between mother and infant during routine painful procedures and parent-led pain alleviation. Since the concept synchrony was not previously clearly described in the manuscript, we have now revised both the Introduction and the Discussion to include a more detailed framing of the concept synchrony and connected brain areas. We hope the rationale is now better motivated. |
| 2. The manuscript should describe the methods in sufficient detail to prevent undisclosed flexibility in the experimental procedure or analysis pipeline, including sufficient outcome-neutral conditions (e.g. necessary controls, absence of floor or ceiling effects) to test the proposed hypotheses and a statistical power analysis where applicable. As there may be aspects of the methodology and analysis which can only be refined once the work is undertaken, authors should outline potential assumptions and explicitly describe what aspects of the proposed analyses, if any, are exploratory. | Revisions are made both in the Introduction, Methods and the Discussion to better describe the hypotheses in the study. |
|  |  |
| Overall, the protocol is technically sound. Nonetheless, I have some reservations regarding some of the procedures.  2.1. Study sample: twenty mother-infant dyads might not be a sufficient sample size for a fNIRS hyperscanning study, especially when involving a clinical population. Considering the high attrition rates in hyperscanning studies with infants (Billeci et al. 2024, doi: 10.3389/fpsyg.2024.1516616), it is recommended to always perform an oversampling (with an increase of at least 50% of the selected sample) to secure the minimum sample size for appropriately powered statistical analyses. The “typical” parent-infant hyperscanning study has an average final sample size of 30-40 dyads. | We have now performed a MESI analysis which gave the sample size of 11. To compensate for attrition, we will recruite 20 dyads. |
| 2.2. The authors should specify the rationale behind measuring interpersonal neural synchrony on frontal and temporal areas. | This comment is very important, and the manuscript is now revised to include a more detailed framing of the concept synchrony and connected brain areas. Revisions are made both in the Introduction and the Discussion |
| 2.3. While it would be interesting and relevant to obtain multiple measurements of neurophysiological synchrony, I am concerned that the experimental procedure as described here may discourage eligible subjects from participating. Parents of such a vulnerable population may perceive the application of multiple neurophysiological data acquisition devices as “too overwhelming” or “too stressful” for the infant, preventing them from participating in the study. In my opinion, the authors should select the minimum number of neurophysiological data acquisition techniques that allow them to test their experimental hypotheses. This would help increase the final sample size as well as reduce the preparation time. | Thank you for raising this concern. We have tried to design the study to minimize the burden on the mothers and infants. We have experience of both the measurements and the combined procedure with skin-to-skin contact, breastfeeding, and lullaby singing. We have tested the setup and data collection in a “dummy” situation (no blood sampling), and it was tolerated well by both the mother and infant. |
| 2.4. The authors should provide more information regarding the plan of statistical analysis, as they only state that they would use parametric statistical tests for normally distributed data and non-parametric tests for data which is not normally distributed. | We have added information about the statistical analyses. |
| 2.5. The authors should clarify the timepoint for the end of recruitment: in which month (approximately) of 2027 will it end? | We have added this information. |
| 3. Descriptions of methods and materials in the protocol should be reported in sufficient detail for another researcher to reproduce all experiments and analyses. The protocol should describe the appropriate controls, sample size calculations, and replication needed to ensure that the data are robust and reproducible. | We have tried to describe this more in detail. |
| The methodology is overall feasible, and the authors have provided a detailed description of the experimental procedure as well as the materials that will be utilized in the proposed study. However, at its current state, the protocol is missing important pieces of information that are fundamental for its reproducibility.  3.1. The protocol does not currently provide a description of how the neurophysiological data acquired via fNIRS will be pre-processed, and does not state which metric will be used to compute interpersonal neural synchrony between mothers and infants. The authors should include these pieces of information in the study and motivate the choice of their pre-processing and synchrony analyses procedures. | We have added information about preprocessing and the outcome variables. |
| 3.2. The protocol should include the inclusion and exclusion criteria for the participants’ enrolment. | We have now defined inclusion and exclusion criteria. |
| 3.3. The authors should describe how they plan on synchronizing neurophysiological data and video recordings. | We have explained this now. |
| 3.4. Point 2 of the procedure timeline (Data analysis paragraph) is unclear. Will you start the neurophysiological data acquisition after the mother has been singing for two minutes, or will you start recording as soon as the mother starts singing and keep recording for two minutes? | This is now detailed in the manuscript. |
| 3.5. Please specify if the neurophysiological data acquisition will be continuous (with keyboard markers signaling the start and end of the specific trials) or separate for each trial. | Yes, we will use keyboard markers and have explained this. |
| 3.6. Please specify what you mean with “in depth analysis of video footage and eye-tracking recordings”. | The Method section is revised, and the microanalysis is now described in more detail. |
| 3.7. Please explain why the fNIRS montage of the infant and the parent (figure 2) is different. | This is explained in the text (16 channels in each cap) |
| 5. The manuscript is written in standard English and requires minimum proofreading for typos. | Thank you, we have performed a proofreading on the reviewed manuscript. |
| **Reviewer #2** |  |
| This submitted protocol addresses a highly relevant and timely topic in neonatal neuroscience and pain research. The proposed integration of fNIRS hyperscanning with physiological measures and detailed interactional microanalysis represents a sophisticated and innovative attempt to “harvest” and understand dyadic neurophysiological mechanisms underlying parent-led multimodal pain alleviation. The focus on synchronous brain activity between mother and infant during routine painful procedures is scientifically compelling and, if successfully implemented, could meaningfully advance both theoretical understanding and clinical practice in neonatal pain management. The overall rationale for the study is clearly articulated. | Thank you! |
| However, when evaluated specifically against the formal PLOS ONE Study Protocol requirements, the manuscript currently presents several substantive omissions and areas of insufficient reporting that must be addressed before it can be considered compliant as a protocol paper:  First, although the manuscript is clearly framed as a Study Protocol and recruitment has not yet commenced, there is no indication of trial registration in a publicly accessible WHO- or ICMJE-approved registry, nor is any registration number provided in the Abstract. Given that this study prospectively assigns behavioral interventions (skin-to-skin contact, breastfeeding, and live singing) during a clinical procedure, it meets PLOS ONE’s operational definition of a clinical trial. Thus, if this classification applies, registration prior to submission is mandatory, and the registry name and identifier must be explicitly reported. In addition, required clinical trial documentation is currently absent, including submission of the ethics-approved protocol as supplementary material, inclusion of a SPIRIT schedule as Figure 1, and provision of a completed SPIRIT checklist. If the authors believe this study does not constitute a clinical trial, this should be explicitly justified in the manuscript. | We do not define this as a clinical trial since we are not testing the efficacy of any intervention but are exploring the brain synchrony during methods already used in clinical settings. Thus, we don’t think it should be registered as a clinical trial. For the same reason the SPIRIT protocol is not used.  However, if so required by the editor, we will register the study. |
| Second, while the Methods section provides a detailed procedural description of the experimental setup and instrumentation, several core protocol elements required by the journal’s guidelines are missing or insufficiently specified. Most notably, no formal sample size or power calculation is presented. The choice of 20 mother–infant dyads is justified only by reference to previous studies, which does not satisfy protocol standards for this research outlet. A quantitative rationale for sample size, based on expected effect sizes or synchrony metrics, is at the minimum required. | We have now performed a MESI analysis which gave the sample size of 11. To compensate for attrition we will recruite 20 dyads. |
| Relatedly, explicit inclusion and exclusion criteria are not defined. The manuscript refers generally to “healthy newborn infants,” but does not operationalize this term (e.g., gestational age range, birth weight thresholds, Apgar scores, maternal characteristics, or medical exclusions). For reproducibility and transparency, these criteria must be clearly stated. | We have now defined inclusion and exclusion criteria. |
| Third, the statistical analysis plan remains overly generic for a protocol paper. While the authors mention the use of parametric and non-parametric tests depending on data distribution, there is no specification of primary versus secondary outcomes, no description of planned synchrony metrics for hyperscanning data, no approach to repeated measures or dyadic dependency, no strategy for handling missing or noisy fNIRS channels, and no indication of correction for multiple comparisons. PLOS ONE protocols require a substantially more detailed and pre-specified analytical framework. | We have expanded the analysis section. |
| Fourth, the manuscript does not include a data management plan. There is no description of how data will be stored, anonymized, governed, or archived, nor how sensitive infant and maternal neurophysiological data will be protected over time. This is a required component of Study Protocol submissions in this journal. | We have added this information. |
| Finally, although a projected study timeline is provided, the current study status is not formally stated (e.g., “not yet recruiting”), and safety considerations are described only briefly. While the interventions are non-pharmacological, PLOS ONE nevertheless expects explicit reporting of monitoring procedures and any anticipated risks, even if minimal. | Thank you, we have added this information. |
| I would suggest the authors to consult as a template the recently published paper by Papatzikis et al., 2025, referring to the same domain of research, on top of the PlOS ONE author guidelines before they resubmit.  Papatzikis, E., Dimitropoulos, K., Tataropoulou, K., Kyrtsoudi, M., Pasoudi, E., O’Toole, J. M., & Nika, A. (2025). The father’s singing voice may impact premature infants’ brain more than their mother’s: A NICU single-arm exploratory study protocol and preliminary data on a singing and EEG framework based on the fundamental frequency of voice and kinship. Plos one, 20(8), e0328211. | Thank you, we found it relevant to include. |
| **Reviewer #3** |  |
| Thank you for the opportunity to review the manuscript titled “Synchronous brain activity in newborn infants and their mothers during parent-led multimodal pain alleviation with skin-to-skin contact, breastfeeding, and lullaby singing.” This study protocol explores a timely and significant area by combining hyperscanning fNIRS with physiological, behavioral, and interactional assessments within a mother–infant setting. The manuscript is well-written and presents an innovative, ecologically valid approach to studying parent-led multimodal pain relief. To enhance the protocol further, several points would benefit from clarification and expansion | Thank you for this and for your suggestions. We have tried to answer them below. |
| Introduction:  1. On page 4, it is stated: “A new, often overlooked and unexplored topic within brain and pain research is multimodal parent-led pain management, which also includes the parents’ live singing.” This is a strong claim regarding the current state of the field. It would be helpful to provide supporting references. | The Introduction is revised and we have also revised this statement. |
| 2. The authors note that recent studies have used eye-tracking to evaluate where observers look during neonatal pain assessment (references 23–26). A brief summary of the key findings of these studies would help contextualize the rationale for including maternal gaze tracking in the present study. | In the Introduction, we have added a summary of the results from these publications. |
| 3. The conceptual link between neural synchrony and analgesic outcomes could be further elaborated. | Absolutely. In the Introduction and Discussion we have now focused more on the conceptual link. |
| 4. The paragraphs introducing heart rate variability (HRV) and skin conductance (SC) appear toward the end of the Introduction, and their placement feels somewhat abrupt in relation to the preceding sections of the Introduction. While these autonomic measures are clearly relevant to the study, the rationale for introducing them at this point could be articulated more explicitly. Strengthening the conceptual linkage to the earlier presentation of neural synchrony and multimodal parent-led pain alleviation, as well as creating a smoother transition into the subsequent Methods section, would improve the overall coherence and flow of the Introduction. | The Introduction has been revised, and these paragraphs have now hopefully been better integrated in the flow of the text. |
| Materials and Methods:  5. The singing component of the multimodal parent-led pain alleviation is described as the mother singing “soft children’s songs” for three minutes. Additional specification of this component would enhance methodological clarity. It would be helpful to clarify whether mothers select their own songs, whether they sing lullabies as indicated at the manuscript heading, whether any guidance is provided, whether repertoire varies across dyads, and whether relevant musical parameters are documented. | The parental live singing intervention is now described in more detail. |
| 6. Given that fNIRS recordings/ assessments are conducted during breastfeeding and during an active clinical procedure, both infant and maternal movement are likely. Clarifying how movement-related artifacts will be monitored and addressed would be relevant in this context. | We anticipate that both the mother and the infant will be fairly still during the data collection but to handle artefacts we will use short-channels and preprocessing of data. We have explained this in the Methods and the Limitations. |
| 7. The planned sample size of 20 mother-infant dyads is justified with reference to prior studies. Providing a sample size calculation, or more explicitly framing the study as exploratory/feasibility-oriented with reference to expected effect sizes, would strengthen the methodological foundation. | We have now performed a MESI analysis which gave the sample size of 11. To compensate for attrition we will recruite 20 dyads. |
| 8. Additional clarification of inclusion and exclusion criteria (e.g., gestational age range, relevant medical factors, hearing problems) would enhance reproducibility and interpretability. | We have now defined inclusion and exclusion criteria. |
| 9. While the hyperscanning fNIRS configuration is clearly described, additional information regarding data preprocessing procedures would enhance methodological transparency. | The preprocessing is now described in the Data analysis section. |
| 10. It would be advisable to explain and specify the methodological framework that will be used for the video microanalysis of mother–infant interaction. Clarifying how the microanalysis will be conducted (e.g., whether a structured coding system will be used, whether analyses will be performed frame-by-frame, how interactional variables are operationalized, and how inter-rater reliability will be ensured) would strengthen methodological transparency. | Thank you for your comment. The microanalysis is now described in detail in the Method section. |
| 11. The description of the statistical analysis is relatively brief. Given the complexity of the hyperscanning design, the dyadic structure of the data, and the multiple measurements, providing a more detailed description of the planned analyses would enhance understanding. | We have added this information. |
| Discussion:  12. The statement “Synchrony is a key feature of mother–infant interactions” would benefit from additional theoretical grounding and references. Given that synchrony constitutes a central conceptual pillar of the study, expanding this section to briefly distinguish between behavioral, physiological, and neural synchrony and situating the concept within established developmental frameworks would strengthen the theoretical foundation of the manuscript. | This comment is very important, and the manuscript is now revised to include a more detailed framing of the concept synchrony. Revisions are made both in the Introduction and the Discussion. |
| 13. The conceptual pathway linking multimodal parent-led pain alleviation, synchronous brain activity, and the physiological and behavioral measures assessed in the study could be articulated more explicitly. | Absolutely, thank you. The conceptual link and the concept synchrony were not clearly described in the manuscript. We have now revised both the Introduction and the Discussion to include a more detailed framing of the concept synchrony and the conceptual link. We hope these parts are clearer now. |
| 14. While the potential scientific clinical implications of the study are important, explicitly framing them as being hypothesized would maintain appropriate caution for a protocol study. | We have modified the manuscript accordingly. |
| 15. Including a paragraph on potential limitations would further strengthen the Discussion. | A paragraph on potential limitations is now added to the Discussion. |
|  |  |
|  |  |
