# Supplementary material for: Synchronous brain activity in newborn infants and their mothers during parent-led multimodal pain alleviation with skin-to-skin contact, breastfeeding and lullaby singing: Study protocol for a fNIRS hyperscanning study
Source: PLoS One. 2026 Jul 16;21(7):e0353856. doi: 10.1371/journal.pone.0353856 (PMC13375031; doi:10.1371/journal.pone.0353856)
Supplement: Attachment — Submitted filename: Response letter.docx [file pone.0353856.s001.docx]

Dear editor,

Thank you for your feedback concerning text duplication. We do understand this, though it is difficult to find new phrasing, especially when having written own texts on the subject.

However, we have gone through the documents you linked to, and tried to rephrase sentences in our text that are similar to the other sources. We have also performed a plagiarism check with Ouriginal which is the system our university uses, and have further tried to avoid any text similarities with other documents.

We hope that you will now find the manuscript for evaluation for PLOS ONE.

Sincerely

/Mats Eriksson

For the authors
