# Supplementary material for: Synchronous brain activity in newborn infants and their mothers during parent-led multimodal pain alleviation with skin-to-skin contact, breastfeeding and lullaby singing: Study protocol for a fNIRS hyperscanning study
Source: PLoS One. 2026 Jul 16;21(7):e0353856. doi: 10.1371/journal.pone.0353856 (PMC13375031; doi:10.1371/journal.pone.0353856)
Supplement: Attachment — Submitted filename: Review of revised manuscript-PlosOne.docx [file pone.0353856.s002.docx]

**Overall evaluation**

The authors have done an excellent job addressing the reviewers’ comments. The manuscript has significantly improved in clarity, structure, and conceptual coherence, and presents a compelling and well-articulated study protocol. The topic is timely, important, and innovative.

In my view, the manuscript is suitable for publication pending very minor revisions, as outlined below.

**Introduction**

I recommend adding an appropriate reference to support the following statement: “Early stressful ruptures of the infant-parent emotional attachment bond… will cause disturbances in the synchronization of the dyad’s interpersonal psychobiological systems.”

Please introduce the abbreviation for skin conductance (SC) at its first occurrence in the text. Currently, it appears to be defined one sentence after it is already used (e.g., around page 8).

**Sample, setting and procedure**

I suggest improving the structure of this section by introducing subheadings. While the content is clear, the section is relatively dense, and additional internal organization would enhance readability and guide the reader more effectively through the procedure.

**Data analysis plan**

Given the scope and complexity of the data analysis plan, I recommend adding subheadings to distinguish between the different analytical components, for example: fNIRS data analysis, Physiological measures (HRV, SCA), Video microanalysis, and Statistical analysis. This would substantially improve clarity and help the reader follow the analytical workflow.

I have no further comments beyond these minor points.
