# Supplementary material for: Synchronous brain activity in newborn infants and their mothers during parent-led multimodal pain alleviation with skin-to-skin contact, breastfeeding and lullaby singing: Study protocol for a fNIRS hyperscanning study
Source: PLoS One. 2026 Jul 16;21(7):e0353856. doi: 10.1371/journal.pone.0353856 (PMC13375031; doi:10.1371/journal.pone.0353856)
Supplement: Attachment — Submitted filename: Response_to_Reviewers_auresp_2.docx [file pone.0353856.s004.docx]

**Response to the editor’s and reviewers’ comments**

Thank you for the positive evaluation of our revised manuscript, and for the helpful comments and suggestions for improvement. We respond to the comments below, and demonstrate hem in the manuscript with track changes.

For the authors

Sincerely

/Mats Eriksson & Alexandra Ullsten

| **Academic Editor** |  |
| --- | --- |
| **1. Sample size and attrition**  The new MESI analysis and the target sample size of 20 dyads are helpful. However, given the reviewer’s concern about high attrition rates in infant hyperscanning studies, it would be useful for readers if you could add one or two sentences in the Methods to justify more explicitly why 20 dyads are considered sufficient in your context. For example, you might briefly refer to any available data on attrition from your own or related infant studies, and clarify the expected final analyzable sample size. | Thank you for this comment. The recruitement in the pilot test has worked well and both the mothers and the infants have tolerated the data recording without problems. We have added a sentence about this. |
| **2. Feasibility and burden of the multi‑modal setup**  Reviewer #1 raised reasonable questions about the feasibility of simultaneously collecting fNIRS, HRV, skin conductance, eye tracking, and video in this clinical setting. In your response you note that you have conducted preliminary testing and that the procedure was tolerable. Please consider adding a short description of this feasibility/pilot experience in the Methods (e.g., number of pilot dyads, main observations) to reassure readers that the planned protocol is realistic in routine practice. | Please see above about the feasability in the pilots. We have added some information in the Methods section. |
| **3. Statistical analysis plan**  You have expanded the description of the hypotheses and analysis methods, which is appreciated. To further increase transparency, please ensure that the Statistical Analysis section clearly specifies:  the primary outcome measures for neural synchrony and physiological responses,  the main analysis approach for hyperscanning data (e.g., which synchrony metrics will be used and at what level; how regions/channels are grouped), and  how multiple comparisons will be handled, at least at the level of general approach (e.g., FDR correction, cluster-based methods).  This can remain at the protocol level (you do not need to pre-specify every exploratory analysis), but making the primary analysis pipeline explicit will help avoid perceived flexibility. | Thank you. We have expanded the section about statistical analyses. |
| **4. Clarity of figures and captions**  The revised figures are much improved, but a few minor clarifications would enhance readability:  Figure 1 (timeline):  Please ensure that each numbered event is explicitly defined in the figure legend and/or in the Methods text, so that the temporal structure of the procedure is immediately clear.  Consider expanding all abbreviations used in the figure (e.g., SCA) at first mention in the legend. | We have added information in the legends and in the text for enhanced clarity.  We have explained the abbreviations in the legend. |
| Figure 2 (optode/electrode layout):  Please clarify in the legend what the red and blue channels represent (e.g., channels included vs not included in synchrony analyses, or different ROIs).  If applicable, briefly explain in the text how these channels map onto the 10-20 system and which regions are considered a priori regions of interest. | Thank you. We have described the regions of interest in the text. Since this is an exploratory study, we have tried to cover as much as possible of the cortex of the mother and the whole head of the infant, to capture potential syncrony. We have added a section about this. |
| **Reviewer #3** |  |
| **Overall evaluation**  The authors have done an excellent job addressing the reviewers’ comments. The manuscript has significantly improved in clarity, structure, and conceptual coherence, and presents a compelling and well-articulated study protocol. The topic is timely, important, and innovative.  In my view, the manuscript is suitable for publication pending very minor revisions, as outlined below. | Thank you! |
| **Introduction**  I recommend adding an appropriate reference to support the following statement: “Early stressful ruptures of the infant-parent emotional attachment bond… will cause disturbances in the synchronization of the dyad’s interpersonal psychobiological systems.” | We have added the reference Schore 2021 [13]. |
| Please introduce the abbreviation for skin conductance (SC) at its first occurrence in the text. Currently, it appears to be defined one sentence after it is already used (e.g., around page 8). | Thank you, we have fixed this. |
| **Sample, setting and procedure**  I suggest improving the structure of this section by introducing subheadings. While the content is clear, the section is relatively dense, and additional internal organization would enhance readability and guide the reader more effectively through the procedure. | Thank you. We have added the subheadings *Study persons* and *Procedure* |
| **Data analysis plan**  Given the scope and complexity of the data analysis plan, I recommend adding subheadings to distinguish between the different analytical components, for example: fNIRS data analysis, Physiological measures (HRV, SCA), Video microanalysis, and Statistical analysis. This would substantially improve clarity and help the reader follow the analytical workflow. | Thank you for this suggestion. We have added subheadings in the section. |
