# Supplementary material for: Synchronous brain activity in newborn infants and their mothers during parent-led multimodal pain alleviation with skin-to-skin contact, breastfeeding and lullaby singing: Study protocol for a fNIRS hyperscanning study
Source: PLoS One. 2026 Jul 16;21(7):e0353856. doi: 10.1371/journal.pone.0353856 (PMC13375031; doi:10.1371/journal.pone.0353856)
Supplement: Peer review history

# Author comment: Author response to Decision Letter 0

**DOI:** 10.1371/journal.pone.0353856.r001

19 Dec 2025

Attachment Submitted filename: Response letter.docx

# Peer review: Decision Letter 0

**DOI:** 10.1371/journal.pone.0353856.r002

**Authors:** Yoko Hasegawa

20 Feb 2026

PONE-D-25-59989

Synchronous brain activity in newborn infants and their mothers during parent-led multimodal pain alleviation with skin-to-skin contact, breastfeeding and lullaby singing. Study protocol for a fNIRS hyperscanning study

PLOS One

Dear Dr. Eriksson,

Thank you for submitting your manuscript to PLOS ONE. After careful consideration, we feel that it has merit but does not fully meet PLOS ONE’s publication criteria as it currently stands. Therefore, we invite you to submit a revised version of the manuscript that addresses the points raised during the review process.

Please submit your revised manuscript by Apr 06 2026 11:59PM. If you will need more time than this to complete your revisions, please reply to this message or contact the journal office at plosone@plos.org. When you’re ready to submit your revision, log on to https://www.editorialmanager.com/pone/ and select the 'Submissions Needing Revision' folder to locate your manuscript file.

Please include the following items when submitting your revised manuscript:

- A letter that responds to each point raised by the academic editor and reviewer(s). You should upload this letter as a separate file labeled 'Response to Reviewers'.
- A marked-up copy of your manuscript that highlights changes made to the original version. You should upload this as a separate file labeled 'Revised Manuscript with Track Changes'.
- An unmarked version of your revised paper without tracked changes. You should upload this as a separate file labeled 'Manuscript'.

If you would like to make changes to your financial disclosure, please include your updated statement in your cover letter. Guidelines for resubmitting your figure files are available below the reviewer comments at the end of this letter.

If applicable, we recommend that you deposit your laboratory protocols in protocols.io to enhance the reproducibility of your results. Protocols.io assigns your protocol its own identifier (DOI) so that it can be cited independently in the future. For instructions see: https://journals.plos.org/plosone/s/submission-guidelines#loc-laboratory-protocols. Additionally, PLOS ONE offers an option for publishing peer-reviewed Lab Protocol articles, which describe protocols hosted on protocols.io. Read more information on sharing protocols at https://plos.org/protocols?utm_medium=editorial-email&utm_source=authorletters&utm_campaign=protocols.

We look forward to receiving your revised manuscript.

Kind regards,

Yoko Hasegawa

Academic Editor

PLOS One

**Journal Requirements:**

When submitting your revision, we need you to address these additional requirements.

1. Please ensure that your manuscript meets PLOS ONE's style requirements, including those for file naming. The PLOS ONE style templates can be found at

https://journals.plos.org/plosone/s/file?id=wjVg/PLOSOne_formatting_sample_main_body.pdf and

https://journals.plos.org/plosone/s/file?id=ba62/PLOSOne_formatting_sample_title_authors_affiliations.pdf

2. In the online submission form you indicate that your data is not available for proprietary reasons and have provided a contact point for accessing this data. Please note that your current contact point is a co-author on this manuscript. According to our Data Policy, the contact point must not be an author on the manuscript and must be an institutional contact, ideally not an individual. Please revise your data statement to a non-author institutional point of contact, such as a data access or ethics committee, and send this to us via return email. Please also include contact information for the third party organization, and please include the full citation of where the data can be found.

3. Your ethics statement should only appear in the Methods section of your manuscript. If your ethics statement is written in any section besides the Methods, please delete it from any other section.

4. Please upload a new copy of Figures 1 and 2 as the detail is not clear. Please follow the link for more information:  https://journals.plos.org/plosone/s/figures

5. If the reviewer comments include a recommendation to cite specific previously published works, please review and evaluate these publications to determine whether they are relevant and should be cited. There is no requirement to cite these works unless the editor has indicated otherwise.

**Additional Editor Comments:**

This research addresses the important and timely themes of neonatal pain management and parent-child neuroscience. Overall, the background to the research is considered valid, and reviewers largely agree that this protocol could make a useful contribution to the field. However, all reviewers have highlighted significant points requiring resolution prior to revision concerning methodological details, justification of sample size, and clarity of several conceptual and analytical aspects.

In light of these comments, we request that the manuscript undergo **Major Revision**. Please prepare a detailed point-by-point response to each reviewer's comment, clearly indicating where and how the manuscript has been amended.

[Note: HTML markup is below. Please do not edit.]

Reviewers' comments:

Reviewer's Responses to Questions

**Comments to the Author**

1. Does the manuscript provide a valid rationale for the proposed study, with clearly identified and justified research questions?

The research question outlined is expected to address a valid academic problem or topic and contribute to the base of knowledge in the field.

Reviewer #1: Partly

Reviewer #2: Yes

Reviewer #3: Yes

**********

2. Is the protocol technically sound and planned in a manner that will lead to a meaningful outcome and allow testing the stated hypotheses?

The manuscript should describe the methods in sufficient detail to prevent undisclosed flexibility in the experimental procedure or analysis pipeline, including sufficient outcome-neutral conditions (e.g. necessary controls, absence of floor or ceiling effects) to test the proposed hypotheses and a statistical power analysis where applicable. As there may be aspects of the methodology and analysis which can only be refined once the work is undertaken, authors should outline potential assumptions and explicitly describe what aspects of the proposed analyses, if any, are exploratory.

Reviewer #1: Yes

Reviewer #2: No

Reviewer #3: Yes

**********

3. Is the methodology feasible and described in sufficient detail to allow the work to be replicable?

Descriptions of methods and materials in the protocol should be reported in sufficient detail for another researcher to reproduce all experiments and analyses. The protocol should describe the appropriate controls, sample size calculations, and replication needed to ensure that the data are robust and reproducible.

Reviewer #1: No

Reviewer #2: No

Reviewer #3: Yes

**********

4. Have the authors described where all data underlying the findings will be made available when the study is complete?

The PLOS Data policy requires authors to make all data underlying the findings described in their manuscript fully available without restriction, with rare exception, at the time of publication. The data should be provided as part of the manuscript or its supporting information, or deposited to a public repository. For example, in addition to summary statistics, the data points behind means, medians and variance measures should be available. If there are restrictions on publicly sharing data—e.g. participant privacy or use of data from a third party—those must be specified.

Reviewer #1: Yes

Reviewer #2: No

Reviewer #3: Yes

**********

5. Is the manuscript presented in an intelligible fashion and written in standard English?

PLOS ONE does not copyedit accepted manuscripts, so the language in submitted articles must be clear, correct, and unambiguous. Any typographical or grammatical errors should be corrected at revision, so please note any specific errors here.

Reviewer #1: Yes

Reviewer #2: Yes

Reviewer #3: Yes

**********

6. Review Comments to the Author

Please use the space provided to explain your answers to the questions above and, if applicable, provide comments about issues authors must address before this protocol can be accepted for publication. You may also include additional comments for the author, including concerns about research or publication ethics.

You may also provide optional suggestions and comments to authors that they might find helpful in planning their study.

(Please upload your review as an attachment if it exceeds 20,000 characters)

Reviewer #1: 1. Does the manuscript provide a valid rational for the proposed study, with clearly identified and justified research questions?

The research question outlined is expected to address a valid academic problem or topic and contribute to the base knowledge in the field.

The study protocol tackles a relevant research question, and the experimental hypotheses are clearly stated. However, while I understand the logic behind the choice of measuring HRV and GSR in this population, the rationale behind the choice of exploring interpersonal neural synchrony during infant pain management remains overall unclear. The authors should better describe the rationale that motivated the inclusion of interpersonal neural synchrony measures in their study.

2. Is the protocol technically sound and planned in a manner that will lead to a meaningful outcome and allow testing the stated hypotheses?

The manuscript should describe the methods in sufficient detail to prevent undisclosed flexibility in the experimental procedure or analysis pipeline, including sufficient outcome-neutral conditions (e.g. necessary controls, absence of floor or ceiling effects) to test the proposed hypotheses and a statistical power analysis where applicable. As there may be aspects of the methodology and analysis which can only be refined once the work is undertaken, authors should outline potential assumptions and explicitly describe what aspects of the proposed analyses, if any, are exploratory.

Overall, the protocol is technically sound. Nonetheless, I have some reservations regarding some of the procedures.

2.1. Study sample: twenty mother-infant dyads might not be a sufficient sample size for a fNIRS hyperscanning study, especially when involving a clinical population. Considering the high attrition rates in hyperscanning studies with infants (Billeci et al. 2024, doi: 10.3389/fpsyg.2024.1516616), it is recommended to always perform an oversampling (with an increase of at least 50% of the selected sample) to secure the minimum sample size for appropriately powered statistical analyses. The “typical” parent-infant hyperscanning study has an average final sample size of 30-40 dyads.

2.2. The authors should specify the rationale behind measuring interpersonal neural synchrony on frontal and temporal areas.

2.3. While it would be interesting and relevant to obtain multiple measurements of neurophysiological synchrony, I am concerned that the experimental procedure as described here may discourage eligible subjects from participating. Parents of such a vulnerable population may perceive the application of multiple neurophysiological data acquisition devices as “too overwhelming” or “too stressful” for the infant, preventing them from participating in the study. In my opinion, the authors should select the minimum number of neurophysiological data acquisition techniques that allow them to test their experimental hypotheses. This would help increase the final sample size as well as reduce the preparation time.

2.4. The authors should provide more information regarding the plan of statistical analysis, as they only state that they would use parametric statistical tests for normally distributed data and non-parametric tests for data which is not normally distributed.

2.5. The authors should clarify the timepoint for the end of recruitment: in which month (approximately) of 2027 will it end?

3. Is the methodology feasible and described in sufficient detail to allow the work to be replicable?

Descriptions of methods and materials in the protocol should be reported in sufficient detail for another researcher to reproduce all experiments and analyses. The protocol should describe the appropriate controls, sample size calculations, and replication needed to ensure that the data are robust and reproducible.

The methodology is overall feasible, and the authors have provided a detailed description of the experimental procedure as well as the materials that will be utilized in the proposed study. However, at its current state, the protocol is missing important pieces of information that are fundamental for its reproducibility.

3.1. The protocol does not currently provide a description of how the neurophysiological data acquired via fNIRS will be pre-processed, and does not state which metric will be used to compute interpersonal neural synchrony between mothers and infants. The authors should include these pieces of information in the study and motivate the choice of their pre-processing and synchrony analyses procedures.

3.2. The protocol should include the inclusion and exclusion criteria for the participants’ enrolment.

3.3. The authors should describe how they plan on synchronizing neurophysiological data and video recordings.

3.4. Point 2 of the procedure timeline (Data analysis paragraph) is unclear. Will you start the neurophysiological data acquisition after the mother has been singing for two minutes, or will you start recording as soon as the mother starts singing and keep recording for two minutes?

3.5. Please specify if the neurophysiological data acquisition will be continuous (with keyboard markers signaling the start and end of the specific trials) or separate for each trial.

3.6. Please specify what you mean with “in depth analysis of video footage and eye-tracking recordings”.

3.7. Please explain why the fNIRS montage of the infant and the parent (figure 2) is different.

4. Have the authors described where all data underlying the findings will be made available when the study is complete?

The PLOS data policy requires authors to make all data underlying the findings described in their manuscript fully available without restriction, with rare exception, at the time of publication. The data should be provided as part of the manuscript or its supporting information, or deposited to a public repository. For example, in addition to summary statistics, the data point behind means, medians and variance measures should be available. If there are restrictions on publicly sharing data – e.g. participant privacy or use of data from a third party – those must be specified.

Not applicable

5. Is the manuscript presented in an intelligible fashion and written in standard English?

PLOS ONE does not copyedit accepted manuscripts, so the language in submitted articles must be clear, correct and unambiguous. Any typographical or grammatical errors should be corrected at revision, so please note any specific errors here.

The manuscript is written in standard English and requires minimum proofreading for typos.

Reviewer #2: This submitted protocol addresses a highly relevant and timely topic in neonatal neuroscience and pain research. The proposed integration of fNIRS hyperscanning with physiological measures and detailed interactional microanalysis represents a sophisticated and innovative attempt to “harvest” and understand dyadic neurophysiological mechanisms underlying parent-led multimodal pain alleviation. The focus on synchronous brain activity between mother and infant during routine painful procedures is scientifically compelling and, if successfully implemented, could meaningfully advance both theoretical understanding and clinical practice in neonatal pain management. The overall rationale for the study is clearly articulated.

However, when evaluated specifically against the formal PLOS ONE Study Protocol requirements, the manuscript currently presents several substantive omissions and areas of insufficient reporting that must be addressed before it can be considered compliant as a protocol paper:

First, although the manuscript is clearly framed as a Study Protocol and recruitment has not yet commenced, there is no indication of trial registration in a publicly accessible WHO- or ICMJE-approved registry, nor is any registration number provided in the Abstract. Given that this study prospectively assigns behavioral interventions (skin-to-skin contact, breastfeeding, and live singing) during a clinical procedure, it meets PLOS ONE’s operational definition of a clinical trial. Thus, if this classification applies, registration prior to submission is mandatory, and the registry name and identifier must be explicitly reported. In addition, required clinical trial documentation is currently absent, including submission of the ethics-approved protocol as supplementary material, inclusion of a SPIRIT schedule as Figure 1, and provision of a completed SPIRIT checklist. If the authors believe this study does not constitute a clinical trial, this should be explicitly justified in the manuscript.

Second, while the Methods section provides a detailed procedural description of the experimental setup and instrumentation, several core protocol elements required by the journal’s guidelines are missing or insufficiently specified. Most notably, no formal sample size or power calculation is presented. The choice of 20 mother–infant dyads is justified only by reference to previous studies, which does not satisfy protocol standards for this research outlet. A quantitative rationale for sample size, based on expected effect sizes or synchrony metrics, is at the minimum required.

Relatedly, explicit inclusion and exclusion criteria are not defined. The manuscript refers generally to “healthy newborn infants,” but does not operationalize this term (e.g., gestational age range, birth weight thresholds, Apgar scores, maternal characteristics, or medical exclusions). For reproducibility and transparency, these criteria must be clearly stated.

Third, the statistical analysis plan remains overly generic for a protocol paper. While the authors mention the use of parametric and non-parametric tests depending on data distribution, there is no specification of primary versus secondary outcomes, no description of planned synchrony metrics for hyperscanning data, no approach to repeated measures or dyadic dependency, no strategy for handling missing or noisy fNIRS channels, and no indication of correction for multiple comparisons. PLOS ONE protocols require a substantially more detailed and pre-specified analytical framework.

Fourth, the manuscript does not include a data management plan. There is no description of how data will be stored, anonymized, governed, or archived, nor how sensitive infant and maternal neurophysiological data will be protected over time. This is a required component of Study Protocol submissions in this journal.

Finally, although a projected study timeline is provided, the current study status is not formally stated (e.g., “not yet recruiting”), and safety considerations are described only briefly. While the interventions are non-pharmacological, PLOS ONE nevertheless expects explicit reporting of monitoring procedures and any anticipated risks, even if minimal.

I would suggest the authors to consult as a template the recently published paper by Papatzikis et al., 2025, referring to the same domain of research, on top of the PlOS ONE author guidelines before they resubmit.

Reference:

Papatzikis, E., Dimitropoulos, K., Tataropoulou, K., Kyrtsoudi, M., Pasoudi, E., O’Toole, J. M., & Nika, A. (2025). The father’s singing voice may impact premature infants’ brain more than their mother’s: A NICU single-arm exploratory study protocol and preliminary data on a singing and EEG framework based on the fundamental frequency of voice and kinship. Plos one, 20(8), e0328211.

Reviewer #3: Thank you for the opportunity to review the manuscript titled “Synchronous brain activity in newborn infants and their mothers during parent-led multimodal pain alleviation with skin-to-skin contact, breastfeeding, and lullaby singing.”

This study protocol explores a timely and significant area by combining hyperscanning fNIRS with physiological, behavioral, and interactional assessments within a mother–infant setting.

The manuscript is well-written and presents an innovative, ecologically valid approach to studying parent-led multimodal pain relief. To enhance the protocol further, several points would benefit from clarification and expansion:

Introduction:

1. On page 4, it is stated: “A new, often overlooked and unexplored topic within brain and pain research is multimodal parent-led pain management, which also includes the parents’ live singing.” This is a strong claim regarding the current state of the field. It would be helpful to provide supporting references.

2. The authors note that recent studies have used eye-tracking to evaluate where observers look during neonatal pain assessment (references 23–26). A brief summary of the key findings of these studies would help contextualize the rationale for including maternal gaze tracking in the present study.

3. The conceptual link between neural synchrony and analgesic outcomes could be further elaborated.

4. The paragraphs introducing heart rate variability (HRV) and skin conductance (SC) appear toward the end of the Introduction, and their placement feels somewhat abrupt in relation to the preceding sections of the Introduction. While these autonomic measures are clearly relevant to the study, the rationale for introducing them at this point could be articulated more explicitly. Strengthening the conceptual linkage to the earlier presentation of neural synchrony and multimodal parent-led pain alleviation, as well as creating a smoother transition into the subsequent Methods section, would improve the overall coherence and flow of the Introduction.

Materials and Methods:

5. The singing component of the multimodal parent-led pain alleviation is described as the mother singing “soft children’s songs” for three minutes. Additional specification of this component would enhance methodological clarity. It would be helpful to clarify whether mothers select their own songs, whether they sing lullabies as indicated at the manuscript heading, whether any guidance is provided, whether repertoire varies across dyads, and whether relevant musical parameters are documented.

6. Given that fNIRS recordings/assessments are conducted during breastfeeding and during an active clinical procedure, both infant and maternal movement are likely. Clarifying how movement-related artifacts will be monitored and addressed would be relevant in this context.

7. The planned sample size of 20 mother-infant dyads is justified with reference to prior studies. Providing a sample size calculation, or more explicitly framing the study as exploratory/feasibility-oriented with reference to expected effect sizes, would strengthen the methodological foundation.

8. Additional clarification of inclusion and exclusion criteria (e.g., gestational age range, relevant medical factors, hearing problems) would enhance reproducibility and interpretability.

9. While the hyperscanning fNIRS configuration is clearly described, additional information regarding data preprocessing procedures would enhance methodological transparency.

10. It would be advisable to explain and specify the methodological framework that will be used for the video microanalysis of mother–infant interaction. Clarifying how the microanalysis will be conducted (e.g., whether a structured coding system will be used, whether analyses will be performed frame-by-frame, how interactional variables are operationalized, and how inter-rater reliability will be ensured) would strengthen methodological transparency.

11. The description of the statistical analysis is relatively brief. Given the complexity of the hyperscanning design, the dyadic structure of the data, and the multiple measurements, providing a more detailed description of the planned analyses would enhance understanding.

Discussion:

12. The statement “Synchrony is a key feature of mother–infant interactions” would benefit from additional theoretical grounding and references. Given that synchrony constitutes a central conceptual pillar of the study, expanding this section to briefly distinguish between behavioral, physiological, and neural synchrony and situating the concept within established developmental frameworks would strengthen the theoretical foundation of the manuscript.

13. The conceptual pathway linking multimodal parent-led pain alleviation, synchronous brain activity, and the physiological and behavioral measures assessed in the study could be articulated more explicitly.

14. While the potential scientific clinical implications of the study are important, explicitly framing them as being hypothesized would maintain appropriate caution for a protocol study.

15. Including a paragraph on potential limitations would further strengthen the Discussion.

**********

7. PLOS authors have the option to publish the peer review history of their article (what does this mean?). If published, this will include your full peer review and any attached files.

If you choose “no”, your identity will remain anonymous but your review may still be made public.

**Do you want your identity to be public for this peer review?**  For information about this choice, including consent withdrawal, please see our Privacy Policy.

Reviewer #1: No

Reviewer #2: No

Reviewer #3: **Yes:** Maayan Salomon-Gimmon

**********

[NOTE: If reviewer comments were submitted as an attachment file, they will be attached to this email and accessible via the submission site. Please log into your account, locate the manuscript record, and check for the action link "View Attachments". If this link does not appear, there are no attachment files.]

To ensure your figures meet our technical requirements, please review our figure guidelines: https://journals.plos.org/plosone/s/figures

You may also use PLOS’s free figure tool, NAAS, to help you prepare publication quality figures: https://journals.plos.org/plosone/s/figures#loc-tools-for-figure-preparation.

NAAS will assess whether your figures meet our technical requirements by comparing each figure against our figure specifications.

# Author comment: Author response to Decision Letter 1

**DOI:** 10.1371/journal.pone.0353856.r003

2 Apr 2026

Thank you for all valuable comments. Please see our Respond to reviewer document.

Attachment Submitted filename: Response to Reviewers.docx

# Peer review: Decision Letter 1

**DOI:** 10.1371/journal.pone.0353856.r004

**Authors:** Yoko Hasegawa, Yoko Hasegawa

11 May 2026

PONE-D-25-59989R1

Synchronous brain activity in newborn infants and their mothers during parent-led multimodal pain alleviation with skin-to-skin contact, breastfeeding and lullaby singing. Study protocol for a fNIRS hyperscanning study

PLOS One

Dear Dr. Eriksson,

Thank you for submitting your manuscript to PLOS ONE. After careful consideration, we feel that it has merit but does not fully meet PLOS ONE’s publication criteria as it currently stands. Therefore, we invite you to submit a revised version of the manuscript that addresses the points raised during the review process.

Please submit your revised manuscript by Jun 25 2026 11:59PM. If you will need more time than this to complete your revisions, please reply to this message or contact the journal office at plosone@plos.org. When you're ready to submit your revision, log on to https://www.editorialmanager.com/pone/ and select the 'Submissions Needing Revision' folder to locate your manuscript file.

Please include the following items when submitting your revised manuscript:

- A letter that responds to each point raised by the academic editor and reviewer(s). You should upload this letter as a separate file labeled 'Response to Reviewers'.
- A marked-up copy of your manuscript that highlights changes made to the original version. You should upload this as a separate file labeled 'Revised Manuscript with Track Changes'.
- An unmarked version of your revised paper without tracked changes. You should upload this as a separate file labeled 'Manuscript'.

If you would like to make changes to your financial disclosure, please include your updated statement in your cover letter. Guidelines for resubmitting your figure files are available below the reviewer comments at the end of this letter.

If applicable, we recommend that you deposit your laboratory protocols in protocols.io to enhance the reproducibility of your results. Protocols.io assigns your protocol its own identifier (DOI) so that it can be cited independently in the future. For instructions see: https://journals.plos.org/plosone/s/submission-guidelines#loc-laboratory-protocols. Additionally, PLOS ONE offers an option for publishing peer-reviewed Lab Protocol articles, which describe protocols hosted on protocols.io. Read more information on sharing protocols at https://plos.org/protocols?utm_medium=editorial-email&utm_source=authorletters&utm_campaign=protocols.

As the corresponding author, your ORCID iD is verified in the submission system and will appear in the published article. PLOS supports the use of ORCID, and we encourage all coauthors to register for an ORCID iD and use it as well. Please encourage your coauthors to verify their ORCID iD within the submission system before final acceptance, as unverified ORCID iDs will not appear in the published article. *Only*  the individual author can complete the verification step; PLOS staff *cannot*  verify ORCID iDs on behalf of authors.

We look forward to receiving your revised manuscript.

Kind regards,

Yoko Hasegawa

Academic Editor

PLOS One

Journal Requirements:

If the reviewer comments include a recommendation to cite specific previously published works, please review and evaluate these publications to determine whether they are relevant and should be cited. There is no requirement to cite these works unless the editor has indicated otherwise.

Please review your reference list to ensure that it is complete and correct. If you have cited papers that have been retracted, please include the rationale for doing so in the manuscript text, or remove these references and replace them with relevant current references. Any changes to the reference list should be mentioned in the rebuttal letter that accompanies your revised manuscript. If you need to cite a retracted article, indicate the article’s retracted status in the References list and also include a citation and full reference for the retraction notice.

Additional Editor Comments :

Overall, the reviewers’ main concerns have been addressed, the rationale for investigating mother?infant neural synchrony is now much clearer, and the protocol is technically sound. I therefore consider that the manuscript is suitable for publication in PLOS ONE after minor revisions, as detailed below.

1�Sample size and attrition

The new MESI analysis and the target sample size of 20 dyads are helpful. However, given the reviewer’s concern about high attrition rates in infant hyperscanning studies, it would be useful for readers if you could add one or two sentences in the Methods to justify more explicitly why 20 dyads are considered sufficient in your context. For example, you might briefly refer to any available data on attrition from your own or related infant studies, and clarify the expected final analyzable sample size.

2�Feasibility and burden of the multi‑modal setup

Reviewer #1 raised reasonable questions about the feasibility of simultaneously collecting fNIRS, HRV, skin conductance, eye tracking, and video in this clinical setting. In your response you note that you have conducted preliminary testing and that the procedure was tolerable. Please consider adding a short description of this feasibility/pilot experience in the Methods (e.g., number of pilot dyads, main observations) to reassure readers that the planned protocol is realistic in routine practice.

3�Statistical analysis plan

You have expanded the description of the hypotheses and analysis methods, which is appreciated. To further increase transparency, please ensure that the Statistical Analysis section clearly specifies:

the primary outcome measures for neural synchrony and physiological responses,

the main analysis approach for hyperscanning data (e.g., which synchrony metrics will be used and at what level; how regions/channels are grouped), and

how multiple comparisons will be handled, at least at the level of general approach (e.g., FDR correction, cluster-based methods).

This can remain at the protocol level (you do not need to pre-specify every exploratory analysis), but making the primary analysis pipeline explicit will help avoid perceived flexibility.

4�Clarity of figures and captions

The revised figures are much improved, but a few minor clarifications would enhance readability:

Figure 1 (timeline):

Please ensure that each numbered event is explicitly defined in the figure legend and/or in the Methods text, so that the temporal structure of the procedure is immediately clear.

Consider expanding all abbreviations used in the figure (e.g., SCA) at first mention in the legend.

Figure 2 (optode/electrode layout):

Please clarify in the legend what the red and blue channels represent (e.g., channels included vs not included in synchrony analyses, or different ROIs).

If applicable, briefly explain in the text how these channels map onto the 10-20 system and which regions are considered a priori regions of interest.

[Note: HTML markup is below. Please do not edit.]

Reviewers' comments:

Reviewer's Responses to Questions

**Comments to the Author**

1. Does the manuscript provide a valid rationale for the proposed study, with clearly identified and justified research questions?

The research question outlined is expected to address a valid academic problem or topic and contribute to the base of knowledge in the field.

Reviewer #3: Yes

**********

2. Is the protocol technically sound and planned in a manner that will lead to a meaningful outcome and allow testing the stated hypotheses?

The manuscript should describe the methods in sufficient detail to prevent undisclosed flexibility in the experimental procedure or analysis pipeline, including sufficient outcome-neutral conditions (e.g. necessary controls, absence of floor or ceiling effects) to test the proposed hypotheses and a statistical power analysis where applicable. As there may be aspects of the methodology and analysis which can only be refined once the work is undertaken, authors should outline potential assumptions and explicitly describe what aspects of the proposed analyses, if any, are exploratory.

Reviewer #3: Yes

**********

3. Is the methodology feasible and described in sufficient detail to allow the work to be replicable?

Descriptions of methods and materials in the protocol should be reported in sufficient detail for another researcher to reproduce all experiments and analyses. The protocol should describe the appropriate controls, sample size calculations, and replication needed to ensure that the data are robust and reproducible.

Reviewer #3: Yes

**********

4. Have the authors described where all data underlying the findings will be made available when the study is complete?

The PLOS Data policy requires authors to make all data underlying the findings described in their manuscript fully available without restriction, with rare exception, at the time of publication. The data should be provided as part of the manuscript or its supporting information, or deposited to a public repository. For example, in addition to summary statistics, the data points behind means, medians and variance measures should be available. If there are restrictions on publicly sharing data—e.g. participant privacy or use of data from a third party—those must be specified.

Reviewer #3: Yes

**********

5. Is the manuscript presented in an intelligible fashion and written in standard English?

PLOS ONE does not copyedit accepted manuscripts, so the language in submitted articles must be clear, correct, and unambiguous. Any typographical or grammatical errors should be corrected at revision, so please note any specific errors here.

Reviewer #3: Yes

**********

6. Review Comments to the Author

Please use the space provided to explain your answers to the questions above and, if applicable, provide comments about issues authors must address before this protocol can be accepted for publication. You may also include additional comments for the author, including concerns about research or publication ethics.

You may also provide optional suggestions and comments to authors that they might find helpful in planning their study.

(Please upload your review as an attachment if it exceeds 20,000 characters)

Reviewer #3: Overall evaluation

The authors have done an excellent job addressing the reviewers’ comments. The manuscript has significantly improved in clarity, structure, and conceptual coherence, and presents a compelling and well-articulated study protocol. The topic is timely, important, and innovative.

In my view, the manuscript is suitable for publication pending very minor revisions, as outlined below.

Introduction

I recommend adding an appropriate reference to support the following statement: “Early stressful ruptures of the infant-parent emotional attachment bond… will cause disturbances in the synchronization of the dyad’s interpersonal psychobiological systems.”

Please introduce the abbreviation for skin conductance (SC) at its first occurrence in the text. Currently, it appears to be defined one sentence after it is already used (e.g., around page 8).

Sample, setting and procedure

I suggest improving the structure of this section by introducing subheadings. While the content is clear, the section is relatively dense, and additional internal organization would enhance readability and guide the reader more effectively through the procedure.

Data analysis plan

Given the scope and complexity of the data analysis plan, I recommend adding subheadings to distinguish between the different analytical components, for example: fNIRS data analysis, Physiological measures (HRV, SCA), Video microanalysis, and Statistical analysis. This would substantially improve clarity and help the reader follow the analytical workflow.

I have no further comments beyond these minor points.

**********

7. PLOS authors have the option to publish the peer review history of their article (what does this mean?). If published, this will include your full peer review and any attached files.

If you choose “no”, your identity will remain anonymous but your review may still be made public.

**Do you want your identity to be public for this peer review?**  For information about this choice, including consent withdrawal, please see our Privacy Policy.

Reviewer #3: **Yes:** Maayan Salomon-Gimmon

**********

[NOTE: If reviewer comments were submitted as an attachment file, they will be attached to this email and accessible via the submission site. Please log into your account, locate the manuscript record, and check for the action link "View Attachments". If this link does not appear, there are no attachment files.]

To ensure your figures meet our technical requirements, please review our figure guidelines: https://journals.plos.org/plosone/s/figures

You may also use PLOS’s free figure tool, NAAS, to help you prepare publication quality figures: https://journals.plos.org/plosone/s/figures#loc-tools-for-figure-preparation.

NAAS will assess whether your figures meet our technical requirements by comparing each figure against our figure specifications.

Attachment Submitted filename: Review of revised manuscript-PlosOne.docx

# Author comment: Author response to Decision Letter 2

**DOI:** 10.1371/journal.pone.0353856.r005

28 May 2026

Thank you for your helpful comments. We have tried to adress them in the revised manuscript.

Attachment Submitted filename: Response_to_Reviewers_auresp_2.docx

# Peer review: Decision Letter 2

**DOI:** 10.1371/journal.pone.0353856.r006

**Authors:** Yoko Hasegawa, Yoko Hasegawa, Yoko Hasegawa

1 Jul 2026

Synchronous brain activity in newborn infants and their mothers during parent-led multimodal pain alleviation with skin-to-skin contact, breastfeeding and lullaby singing. Study protocol for a fNIRS hyperscanning study

PONE-D-25-59989R2

Dear Dr. Eriksson,

We’re pleased to inform you that your manuscript has been judged scientifically suitable for publication and will be formally accepted for publication once it meets all outstanding technical requirements.

Within one week, you’ll receive an e-mail detailing the required amendments. When these have been addressed, you’ll receive a formal acceptance letter and your manuscript will be scheduled for publication.

An invoice will be generated when your article is formally accepted. Please note, if your institution has a publishing partnership with PLOS and your article meets the relevant criteria, all or part of your publication costs will be covered. Please make sure your user information is up-to-date by logging into Editorial Manager at Editorial Manager® and clicking the ‘Update My Information' link at the top of the page. For questions related to billing, please contact billing support.

If your institution or institutions have a press office, please notify them about your upcoming paper to help maximize its impact. If they’ll be preparing press materials, please inform our press team as soon as possible -- no later than 48 hours after receiving the formal acceptance. Your manuscript will remain under strict press embargo until 2 pm Eastern Time on the date of publication. For more information, please contact onepress@plos.org.

Kind regards,

Yoko Hasegawa

Academic Editor

PLOS One

Additional Editor Comments (optional):

Reviewers' comments:

Reviewer's Responses to Questions

**Comments to the Author**

1. Does the manuscript provide a valid rationale for the proposed study, with clearly identified and justified research questions?

The research question outlined is expected to address a valid academic problem or topic and contribute to the base of knowledge in the field.

Reviewer #3: Yes

**********

2. Is the protocol technically sound and planned in a manner that will lead to a meaningful outcome and allow testing the stated hypotheses?

The manuscript should describe the methods in sufficient detail to prevent undisclosed flexibility in the experimental procedure or analysis pipeline, including sufficient outcome-neutral conditions (e.g. necessary controls, absence of floor or ceiling effects) to test the proposed hypotheses and a statistical power analysis where applicable. As there may be aspects of the methodology and analysis which can only be refined once the work is undertaken, authors should outline potential assumptions and explicitly describe what aspects of the proposed analyses, if any, are exploratory.

Reviewer #3: Yes

**********

3. Is the methodology feasible and described in sufficient detail to allow the work to be replicable?

Descriptions of methods and materials in the protocol should be reported in sufficient detail for another researcher to reproduce all experiments and analyses. The protocol should describe the appropriate controls, sample size calculations, and replication needed to ensure that the data are robust and reproducible.

Reviewer #3: Yes

**********

4. Have the authors described where all data underlying the findings will be made available when the study is complete?

The PLOS Data policy requires authors to make all data underlying the findings described in their manuscript fully available without restriction, with rare exception, at the time of publication. The data should be provided as part of the manuscript or its supporting information, or deposited to a public repository. For example, in addition to summary statistics, the data points behind means, medians and variance measures should be available. If there are restrictions on publicly sharing data—e.g. participant privacy or use of data from a third party—those must be specified.

Reviewer #3: Yes

**********

5. Is the manuscript presented in an intelligible fashion and written in standard English?

PLOS ONE does not copyedit accepted manuscripts, so the language in submitted articles must be clear, correct, and unambiguous. Any typographical or grammatical errors should be corrected at revision, so please note any specific errors here.

Reviewer #3: Yes

**********

6. Review Comments to the Author

Please use the space provided to explain your answers to the questions above and, if applicable, provide comments about issues authors must address before this protocol can be accepted for publication. You may also include additional comments for the author, including concerns about research or publication ethics.

You may also provide optional suggestions and comments to authors that they might find helpful in planning their study.

(Please upload your review as an attachment if it exceeds 20,000 characters)

Reviewer #3: Thank you for the opportunity to review this revised manuscript. The authors have addressed the previous comments thoughtfully. I believe that this interesting and important manuscript is now suitable for publication.

I congratulate the authors on their valuable work and wish them success with its publication.

**********

7. PLOS authors have the option to publish the peer review history of their article (what does this mean?). If published, this will include your full peer review and any attached files.

If you choose “no”, your identity will remain anonymous but your review may still be made public.

**Do you want your identity to be public for this peer review?**  For information about this choice, including consent withdrawal, please see our Privacy Policy.

Reviewer #3: **Yes:** Maayan Salomon-Gimmon

**********

# Editor report: Acceptance letter

**DOI:** 10.1371/journal.pone.0353856.r007

**Authors:** Yoko Hasegawa, Yoko Hasegawa, Yoko Hasegawa

PONE-D-25-59989R2

PLOS One

Dear Dr. Eriksson,

I'm pleased to inform you that your manuscript has been deemed suitable for publication in PLOS One. Congratulations! Your manuscript is now being handed over to our production team.

At this stage, our production department will prepare your paper for publication. This includes ensuring the following:

* All references, tables, and figures are properly cited

* All relevant supporting information is included in the manuscript submission,

* There are no issues that prevent the paper from being properly typeset

You will receive further instructions from the production team, including instructions on how to review your proof when it is ready. Please keep in mind that we are working through a large volume of accepted articles, so please give us a few days to review your paper and let you know the next and final steps.

Lastly, if your institution or institutions have a press office, please let them know about your upcoming paper now to help maximize its impact. If they'll be preparing press materials, please inform our press team within the next 48 hours. Your manuscript will remain under strict press embargo until 2 pm Eastern Time on the date of publication. For more information, please contact onepress@plos.org.

You will receive an invoice from PLOS for your publication fee after your manuscript has reached the completed accept phase. If you receive an email requesting payment before acceptance or for any other service, this may be a phishing scheme. Learn how to identify phishing emails and protect your accounts at https://explore.plos.org/phishing.

If we can help with anything else, please email us at customercare@plos.org.

Thank you for submitting your work to PLOS ONE and supporting open access.

Kind regards,

PLOS ONE Editorial Office Staff

on behalf of

Dr. Yoko Hasegawa

Academic Editor

PLOS One
